# Supplementary material for: A Cdc42/RhoA regulatory circuit downstream of glycoprotein Ib guides transendothelial platelet biogenesis
Source: Nat Commun. 2017 Jun 15;8:15838. doi: 10.1038/ncomms15838 (PMC5481742; doi:10.1038/ncomms15838)
Supplement: Supplementary Information [file ncomms15838-s1.pdf]

Type of file: PDF
Size of file: 0 KB
Title of file for HTML: Supplementary Information
Description: Supplementary Figures.

Type of file: MP4
Size of file: 0 KB
Title of file for HTML: Supplementary Movie 1
Description: Proplatelet formation of wt MKs.

Type of file: MP4
Size of file: 0 KB
Title of file for HTML: Supplementary Movie 2
Description: Transmigration of $RhoA^{-/-}$ MKs.

Type of file: AVI
Size of file: 0 KB
Title of file for HTML: Supplementary Movie 3
Description: Intrasinusoidal adhesion of $RhoA^{-/-}$ MKs (1).

Type of file: MP4
Size of file: 0 KB
Title of file for HTML: Supplementary Movie 4
Description: Intrasinusoidal adhesion of $RhoA^{-/-}$ MKs (2).

Type of file: AVI
Size of file: 0 KB
Title of file for HTML: Supplementary Movie 5
Description: Release of large proplatelet-like fragments of $RhoA^{-/-}$ MKs.

Type of file: AVI
Size of file: 0 KB
Title of file for HTML: Supplementary Movie 6
Description: MK accumulation and reduced proplatelet formation of $RhoA/Cdc42^{-/-}$ MKs.

Type of file: PDF
Size of file: 0 KB
Title of file for HTML: Peer Review File
Description:

## SUPPLEMENTARY INFORMATION

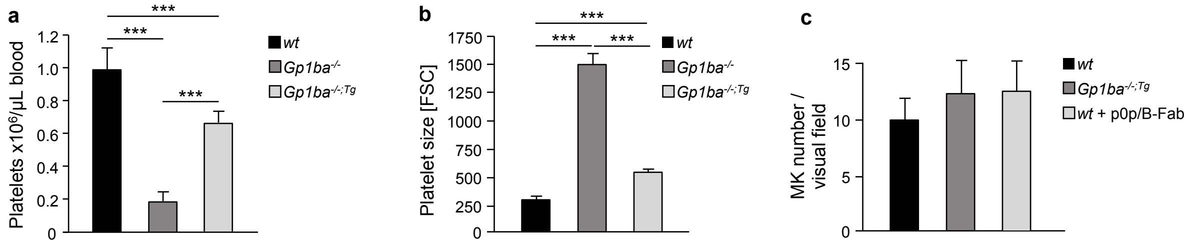


**Supplementary Fig. 1. Macrothrombocytopenia in mice lacking functional GPIbα ectodomain. a,** Reduced peripheral platelet count and **b,** increased platelet size in *Gp1ba*$^{-/-}$ (dark gray) and *Gp1ba*$^{-/-;Tg}$ (*Gp1ba-Tg,* light gray) mice compared to the *wt* (black) determined by flow cytometric analysis (n=4, 5, and 7). Results are representative of 3 independent experiments. **c,** Determination of MK numbers in immunolabeled BM sections from *wt* (black), *Gp1ba*$^{-/-;Tg}$ (dark gray) and *wt* mice after treatment with GPIbα-blocking monovalent Fab fragments, p0p/B-Fab (light gray) (n=4, 7, 5). Bar graphs represent mean ± SD. Two-way ANOVA with Bonferroni correction for multiple comparisons; ***$P$ < 0.001.

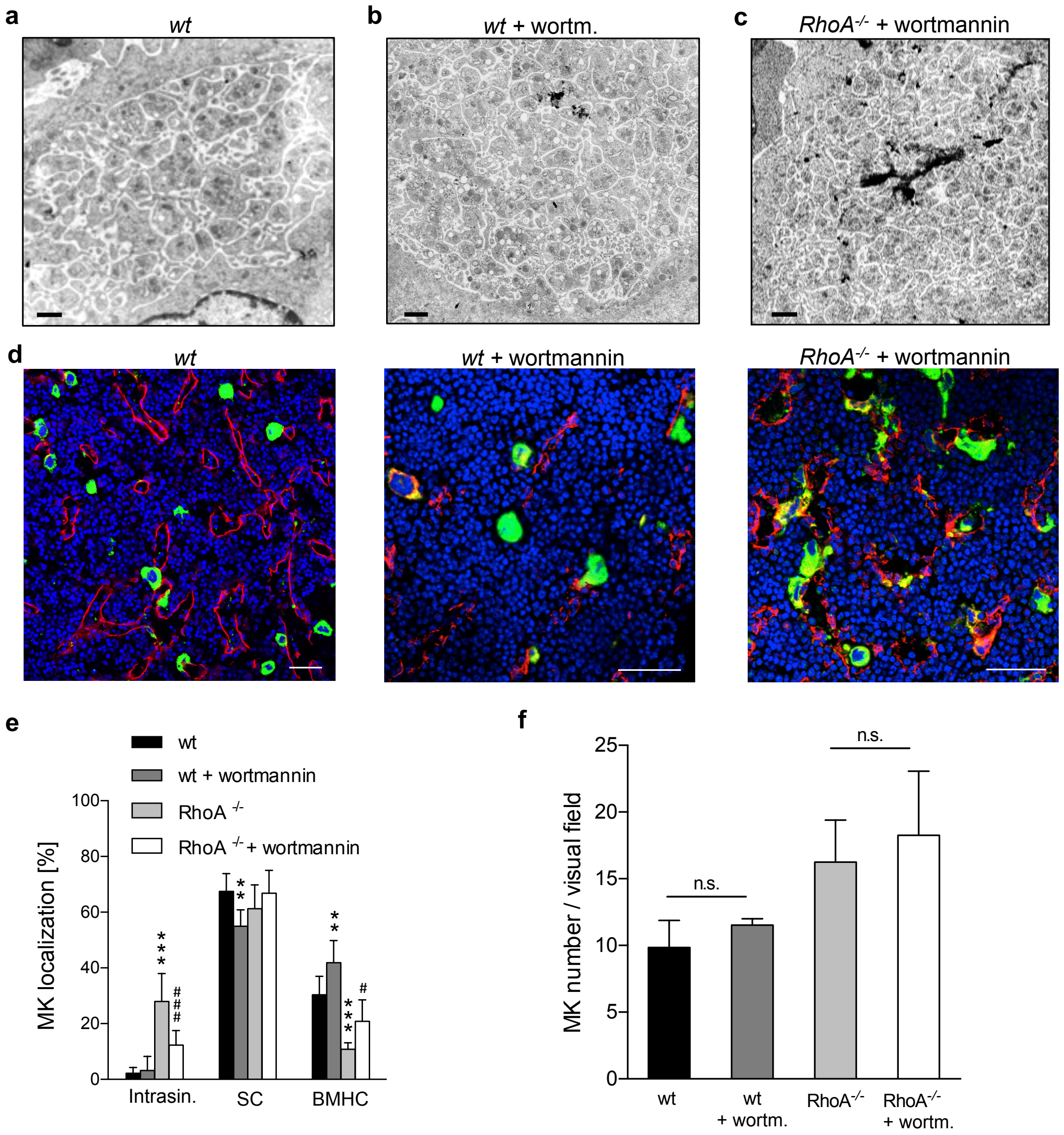


**Supplementary Fig. 2. PI3K inhibition by wortmannin alters MK localization in the BM. a-c,** TEM analysis of BM MKs of *wt* mice (**a**), and *wt* as well as *RhoA*$^{-/-}$ mice after treatment with the PI3K inhibitor wortmannin (**b,c**). Scale bars, 2 µm (**a-c**). **d,** Representative confocal images of immunostained BM MKs of *wt* mice and *wt* as well as *RhoA*$^{-/-}$ mice after treatment with wortmannin. Results are representative of 2 independent experiments. Scale bars, 50 µm. MKs, proplatelets and platelets are shown by GPIX staining in green. Endoglin staining (red) labels vessels. DAPI, blue. **e,** Quantification of MK localization in the BM reveals reduced sinusoidal contact (SC) in *wt* mice (dark gray) and reduced intrasinusoidal localization of *RhoA*$^{-/-}$ MKs (white) upon wortmannin treatment compared to the *wt* (black)

and untreated *RhoA*$^{-/-}$ mice (light gray), respectively (n=5, 6, 10, and 9). Results are pooled from 2 independent experiments. **f,** Determination of MK numbers in immunolabeled BM sections from wt and *RhoA*$^{-/-}$ mice, as well as *wt and RhoA*$^{-/-}$ mice after treatment with wortmannin (n=3, 5, 9, 6). Bar graphs represent mean ± SD. Two-way ANOVA with Bonferroni correction for multiple comparisons; **$P < 0.01$; ***$P < 0.001$ compared to *wt*; $^{\#}P < 0.05$; $^{\#\#\#}P < 0.001$ compared to *RhoA*$^{-/-}$. n.s., not significant.

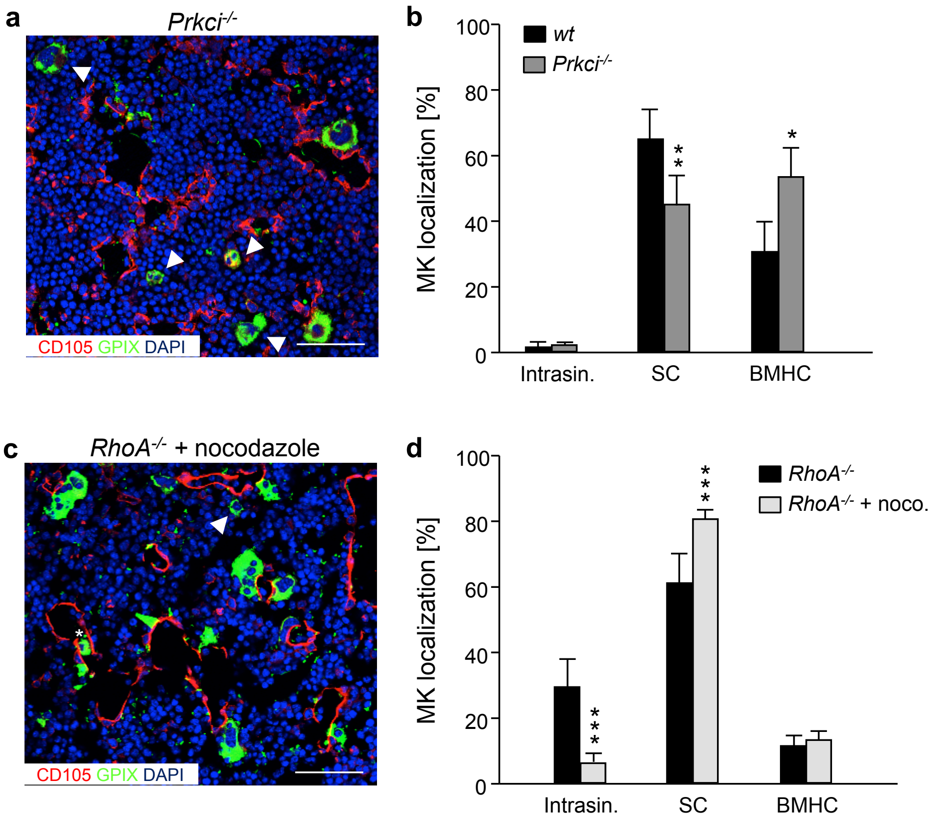


**Supplementary Fig. 3. PKCι-deficiency reduces sinusoidal contact of BM MKs and microtubule disruption reverts intrasinusoidal localization of *RhoA*$^{-/-}$ MKs. a,** Representative confocal images of immunostained BM of *Prcki*$^{-/-}$ mice. Scale bar, 50 µm. MKs, proplatelets and platelets are shown by GPIX staining in green color. Endoglin staining (red) labels vessels. DAPI, blue. Arrowhead indicates MKs in BMHC. **b,** Quantification of MK localization in the BM of wt (black) and *Prcki*$^{-/-}$ mice (dark gray) (n=4 and 7). **c,** Representative confocal image of immunostained BM of *RhoA*$^{-/-}$ mice after treatment with nocodazole. Scale bar, 50 µm. Arrowhead indicates MKs in BMHC, asterisk indicates intrasinusoidal (intrasin.) MKs. **d,** Quantification of MK localization in the BM of *RhoA*$^{-/-}$ mice (black) and nocodazole-treated *RhoA*$^{-/-}$ mice (light gray) (n=4). Bar graphs represent mean ± SD. Two-way ANOVA with Bonferroni correction for multiple comparisons; ***$P$ < 0.001, **$P$ < 0.01, *$P$ < 0.05.

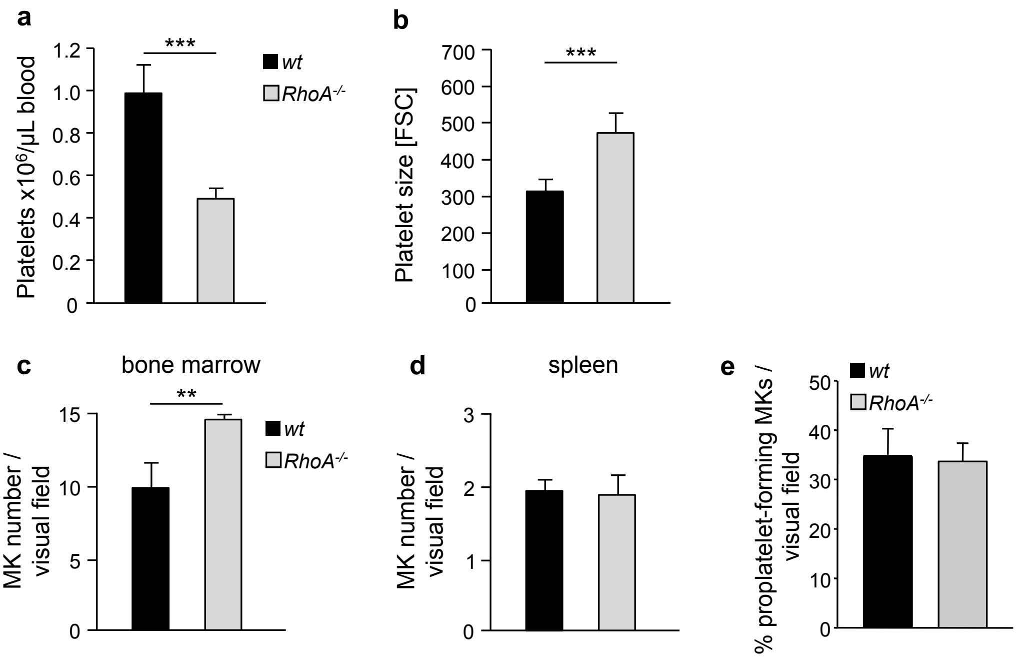


**Supplementary Fig. 4. Macrothrombocytopenia and increased MK number in the BM of *RhoA$^{-/-}$* mice. a,** Reduced peripheral platelet count and **b,** increased platelet size in *RhoA$^{-/-}$* mice (light gray) compared to the *wt* (black), as determined by flow cytometric analysis (n=7 and 5). Results are representative of 5 independent experiments. **c, d,** Determination of MK numbers in hematoxylin and eosin–stained BM (**c**) and spleen (**d**) sections from *wt (black)* and *RhoA$^{-/-}$* mice (light gray) (n=4 and 7). Bar graphs represent mean ± SD. Unpaired two-tailed Student's *t*-test; $^{**}P < 0.01$; $^{***}P < 0.001$.

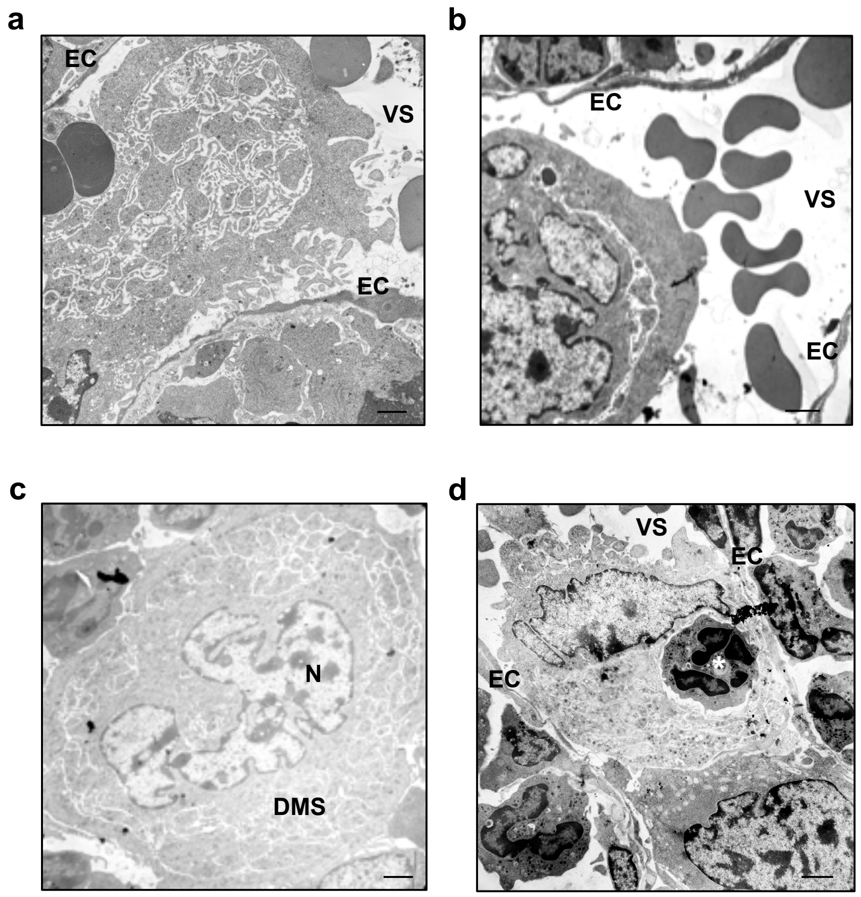


**Supplementary Fig. 5. Normal DMS formation, but intrasinusoidal localization of *RhoA*$^{-/-}$ MKs in the BM. a, b,** TEM analysis of *RhoA*$^{-/-}$ BM MKs reveals MKs adherent to endothelial cells (ECs) inside BM sinusoids (**a**) containing poly-lobulated nuclei (**b**). **c,** DMS formation was unaltered in *RhoA*$^{-/-}$ MKs. **d,** MK showing emperipolesis (indicated by asterisk; n=8). Scale bars, 2.5 µm. EC, endothelial cell; VS, vascular sinus; N, nucleus; DMS, demarcation membrane system.

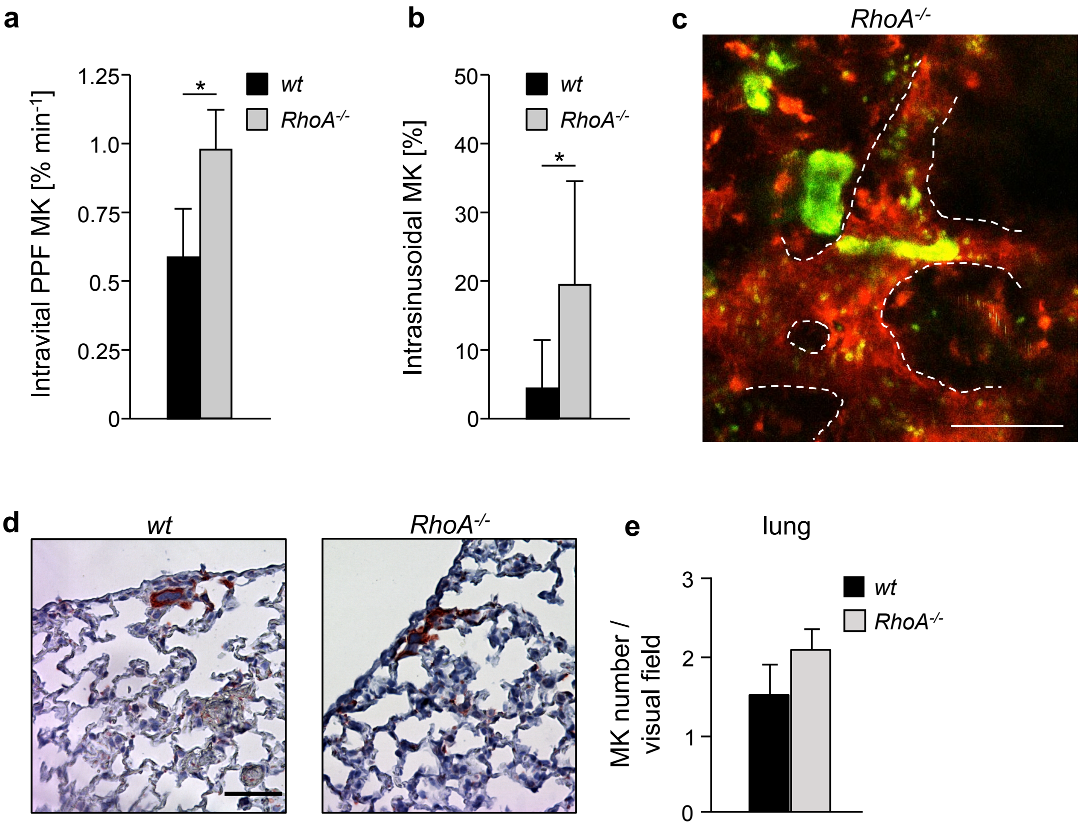


**Supplementary Fig. 6. Increased proplatelet-formation and intrasinusoidal localization of *RhoA*$^{-/-}$ MKs. a,** Quantification of proplatelet-forming (PPF) MKs per minute and **b,** Quantification of intrasinusoidal MKs in *wt* (black) and *RhoA*$^{-/-}$ (light gray) mice (n=7 and 4). **c,** Representative image showing intravital two-photon microscopy of BM *RhoA*$^{-/-}$ MKs in the skull. Scale bars, 50 µm. **d,** Histological analysis of lungs from *wt* (left) and *RhoA*$^{-/-}$ (right) mice. MKs are shown by GPIbα-HRP staining, sections were counterstained with hematoxylin. Scale bar, 50 µm. **e,** Quantification of MK number in *wt* (black) and *RhoA*$^{-/-}$ (light gray) mice in the lung (n=4 and 5). Bar graphs represent mean ± SD. Unpaired two-tailed Student's *t*-test; $^*P < 0.05$.

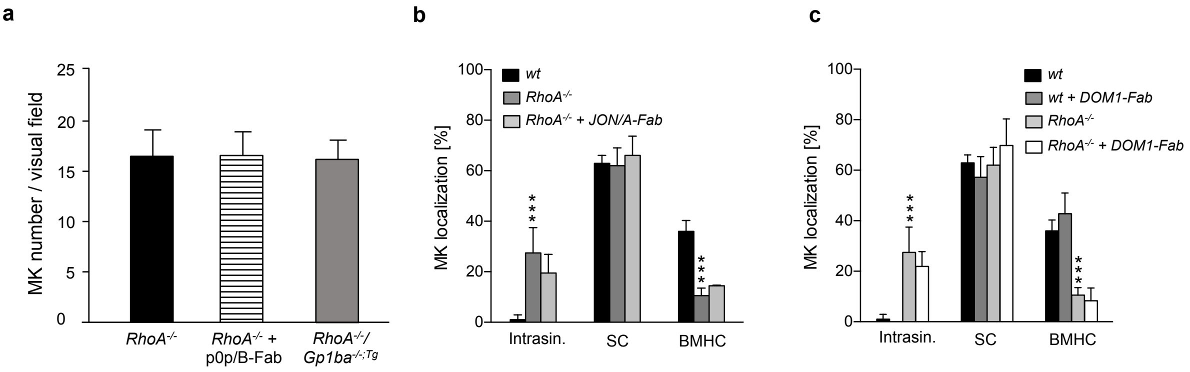


**Supplementary Fig. 7. MK number in the BM of *$RhoA^{-/-}$* mice after GPIbα blockade and MK localization in *wt* and *$RhoA^{-/-}$* mice after treatment with Fab fragments directed against integrin αIIbβ3 and GPV. a,** Unaltered MK number in the BM of *$RhoA^{-/-}$* mice after treatment with GPIbα-blocking monovalent Fab fragments, p0p/B-Fab (light gray), or upon concomitant lack of the GPIbα ectodomain (*$RhoA^{-/-}$/$Gp1ba^{-/-;Tg}$*; dark gray) compared to the *wt* (black). n=8, 3, and 9). **b, c,** Quantification of MK localization in the BM of *wt* (black) and *$RhoA^{-/-}$* mice (light gray) after treatment with monovalent Fab fragments directed against integrin αIIbβ3 (JON/A) **(b)** or GPV (DOM1) **(c)** (n=3, 1 experiment). The vehicle-treated *wt* is shown in black. Bar graphs represent mean ± SD. Two-way ANOVA with Bonferroni correction for multiple comparisons; ***$P < 0.001$.

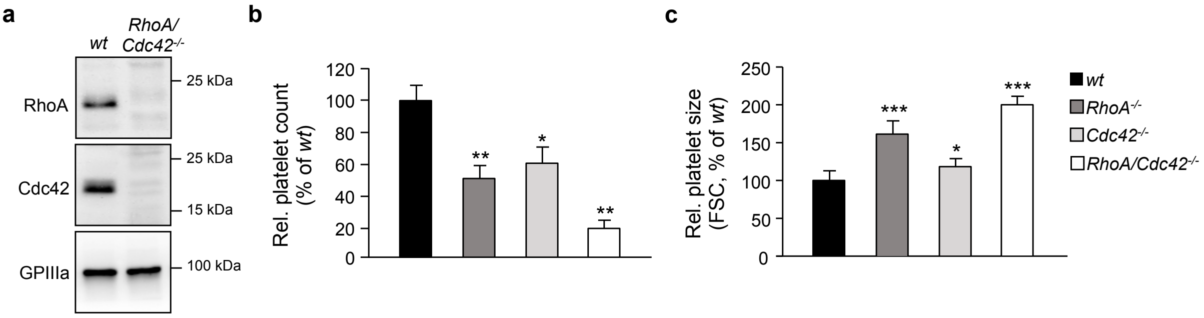


**Supplementary Fig. 8. Severe macrothrombocytopenia in the absence of RhoA and Cdc42 in MKs. a,** Western blot analysis of RhoA and Cdc42 expression in *wt* and *RhoA/Cdc42*$^{-/-}$ platelets. GPIIIa expression was used as loading control. Results shown are representative of 3 independent experiments. **b, c,** Analysis of peripheral platelet count (**b**) and size (**c**) in *wt* (black), *RhoA*$^{-/-}$ (dark gray), *Cdc42*$^{-/-}$ (light gray), and *RhoA/Cdc42*$^{-/-}$ (white) mice (n=4). Results shown are representative of 3 independent experiments. Bar graphs represent mean ± SD. Two-way ANOVA with Bonferroni correction for multiple comparisons; *$P < 0.05$; **$P < 0.01$; ***$P < 0.001$.

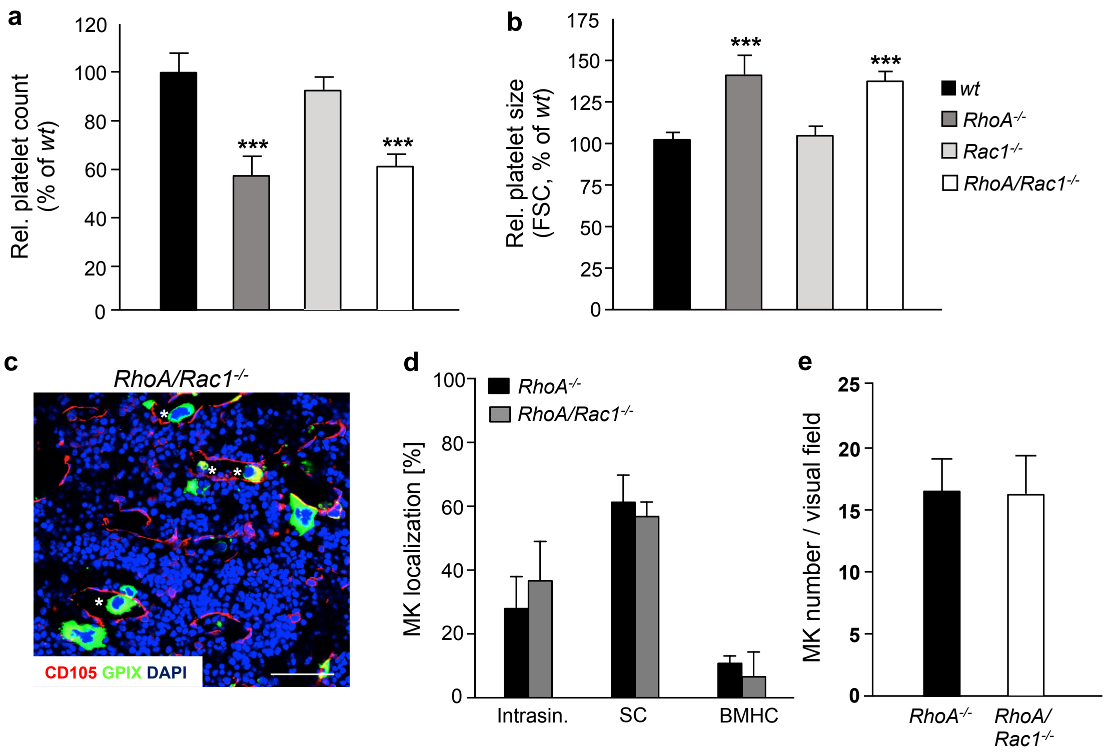


**Supplementary Fig. 9. Concomitant loss of Rac1 does not influence megakaryopoiesis in *RhoA*$^{-/-}$ mice. a, b,** Analysis of peripheral platelet count (**b**) and size (**c**) in *wt* (black), *RhoA*$^{-/-}$ (dark gray), *Rac1*$^{-/-}$ (light gray), and *RhoA/Rac1*$^{-/-}$ (white) mice (n=4). Results are representative of 3 independent experiments. **c,** Representative confocal images of immunostained BM of *RhoA/Rac1*$^{-/-}$ mice. MKs, proplatelets and platelets are shown by GPIX staining in green color. Endoglin staining (red) labels vessels. DAPI, blue. Asterisk indicates intrasinusoidal (intrasin.). Scale bar, 50 µm. **d,** Quantification of MK localization in the BM of *wt* (black) and *RhoA/Rac1*$^{-/-}$ (dark gray) mice (n=9 and 3). **e,** Determination of MK numbers in hematoxylin and eosin–stained BM of *RhoA*$^{-/-}$ (black) and *RhoA/Rac1*$^{-/-}$ (white) mice. Bar graphs represent mean ± SD. Two-way ANOVA with Bonferroni correction for multiple comparisons; ***$P < 0.001$.

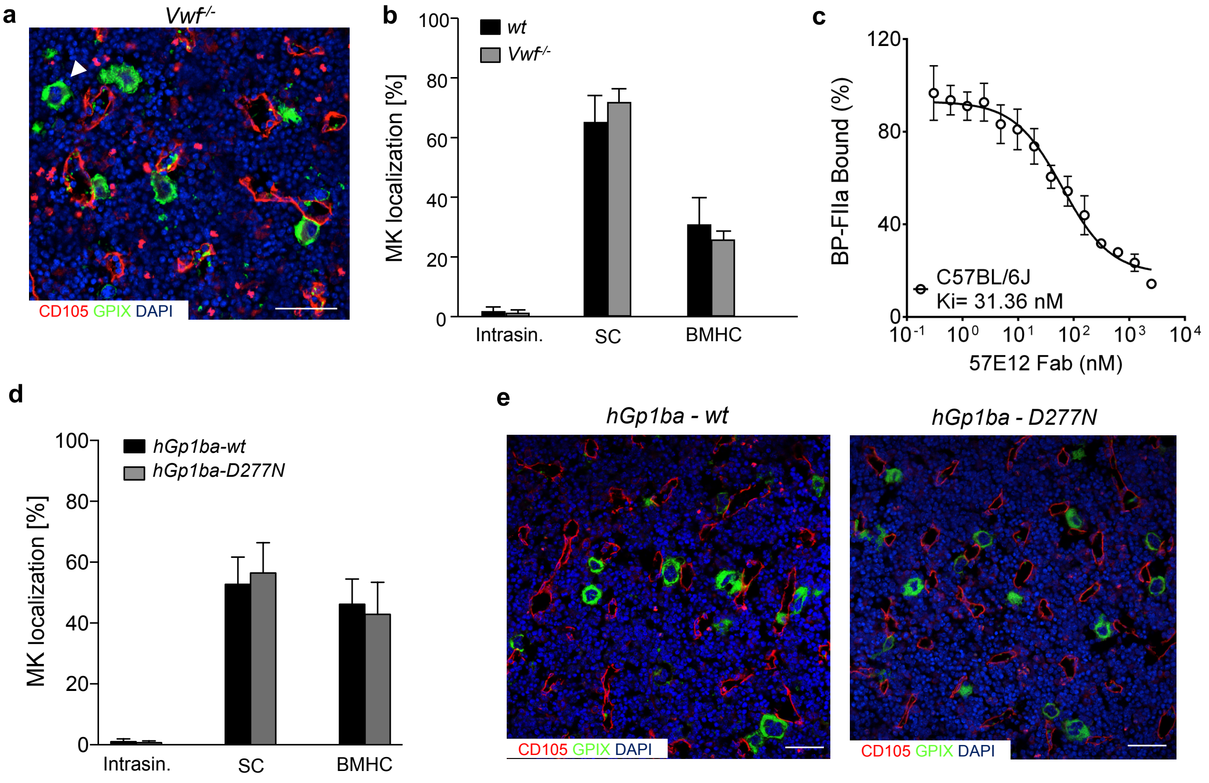


**Supplementary Fig. 10. Deficiency of vWF or mutation of the thrombin binding site in mice expressing human GP1bα does not affect the localization of BM MKs. a,** Representative confocal images of immunostained BM of *Vwf*$^{-/-}$ mice. Scale bar, 50 µm. MKs, proplatelets and platelets are shown by GPIX staining in green color. Endoglin staining (red) labels vessels. DAPI, blue. Arrowhead indicates MKs in BMHC. **b,** Quantification of MK localization in the BM of wt (black) and *Vwf*$^{-/-}$ (dark gray) mice (n=4 and 5). **c,** Monovalent p0p/B-Fab fragments inhibit binding of thrombin (FIIa) to GPIbα. **d,** MK localization in the BM is similar in mice expressing wt (*hGp1ba-wt*) or mutant (*hGp1ba-D277N*) GPIbα unable to bind thrombin (n=3). **e**, Representative confocal images of immunostained BM of transgenic mice harboring *hGp1b-wt* or *hGp1ba-D277N*. Scale bars, 50 µm. MKs, proplatelets and platelets are shown by GPIX staining in green color. Endoglin staining (red) labels vessels. DAPI, blue. Bar graphs represent mean ± SD.

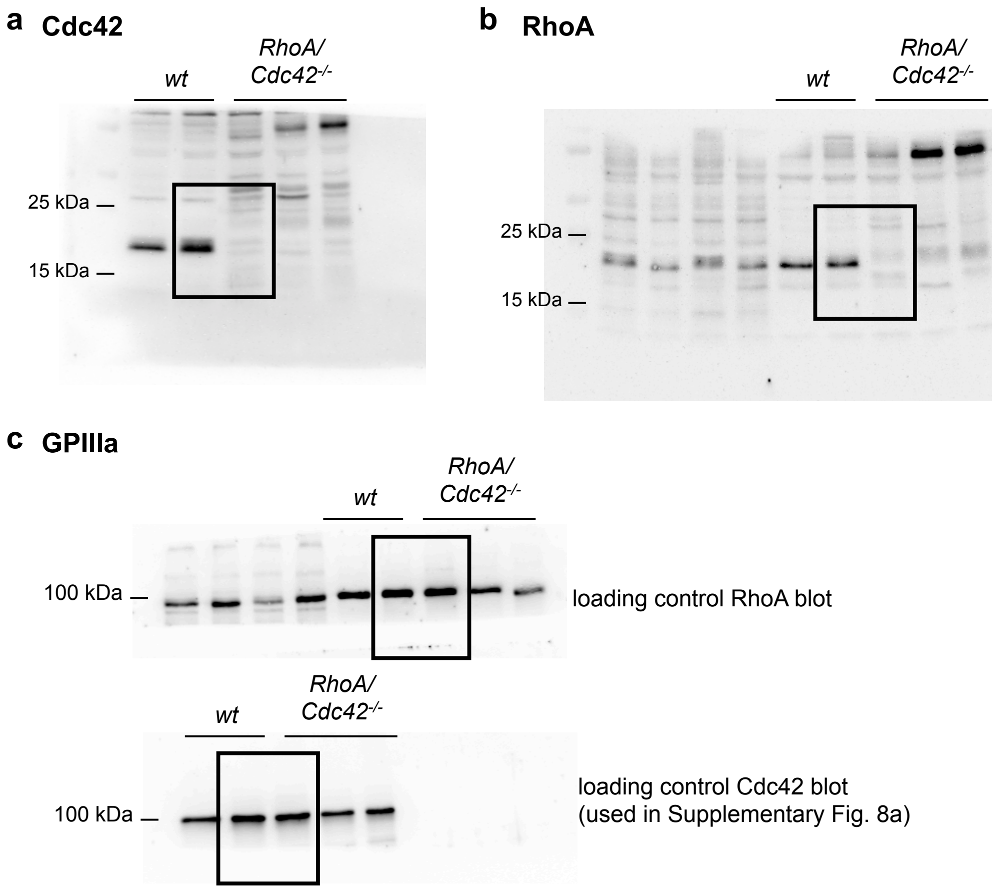


**Supplementary Fig. 11. Uncropped Western blot images.** Western blot analysis of Cdc42 (**a**), RhoA (**b**) and GPIIIa (loading control, **c**) expression in platelet lysates from *wt* and *RhoA/Cdc42*$^{-/-}$ mice. Cropped versions of these images are shown in Supplementary Fig. 8a. Genotypes, molecular weight markers, and selected lanes are depicted.
